# Thyroid hormone activation of retinoic acid synthesis in hypothalamic tanycytes

**DOI:** 10.1002/glia.22938

**Published:** 2015-11-03

**Authors:** Patrick N. Stoney, Gisela Helfer, Diana Rodrigues, Peter J. Morgan, Peter McCaffery

**Affiliations:** ^1^ School of Medical Sciences, Institute of Medical Sciences, University of AberdeenForesterhillAberdeenScotlandAB25 2ZDUnited Kingdom; ^2^ Rowett Institute of Nutrition and Health, University of AberdeenBucksburnAberdeenScotlandAB21 9SBUnited Kingdom

**Keywords:** thyroid hormone receptor, thyroid‐stimulating hormone, deiodinase, retinoic acid receptor, growth

## Abstract

Thyroid hormone (TH) is essential for adult brain function and its actions include several key roles in the hypothalamus. Although TH controls gene expression via specific TH receptors of the nuclear receptor class, surprisingly few genes have been demonstrated to be directly regulated by TH in the hypothalamus, or the adult brain as a whole. This study explored the rapid induction by TH of *retinaldehyde dehydrogenase 1* (*Raldh1*), encoding a retinoic acid (RA)‐synthesizing enzyme, as a gene specifically expressed in hypothalamic tanycytes, cells that mediate a number of actions of TH in the hypothalamus. The resulting increase in RA may then regulate gene expression via the RA receptors, also of the nuclear receptor class. *In vivo* exposure of the rat to TH led to a significant and rapid increase in hypothalamic *Raldh1* within 4 hours. That this may lead to an *in vivo* increase in RA is suggested by the later induction by TH of the RA‐responsive gene *Cyp26b1*. To explore the actions of RA in the hypothalamus as a potential mediator of TH control of gene regulation, an *ex vivo* hypothalamic rat slice culture method was developed in which the *Raldh1*‐expressing tanycytes were maintained. These slice cultures confirmed that TH did not act on genes regulating energy balance but could induce *Raldh1*. RA has the potential to upregulate expression of genes involved in growth and appetite, *Ghrh* and *Agrp*. This regulation is acutely sensitive to epigenetic changes, as has been shown for TH action *in vivo*. These results indicate that sequential triggering of two nuclear receptor signalling systems has the capability to mediate some of the functions of TH in the hypothalamus. GLIA 2016;64:425–439

**Abbreviations**
Agrp: Agouti‐related protein
ARC: Arcuate
DMN: Dorsomedial
HDAC: Histone deacetylase
PFA: Paraformaldehyde
PVN: Paraventricular
Pomc: Proopiomelanocortin
Raldh1: Retinaldehyde dehydrogenase 1
RARs: Retinoic acid receptors
TH: Thyroid hormone
TSH: Thyroid‐stimulating hormone
VMN: Ventromedial

## Introduction

Thyroid hormones (TH) act to control gene expression by binding and activating specific nuclear receptor family transcription factors. TH is known to regulate energy balance and metabolism, with actions in both peripheral tissues and in the brain (reviewed by Lechan and Fekete, 2006; Lopez et al., 2013). Increased food intake (hyperphagia) is a characteristic symptom of hyperthyroidism, the overproduction of TH. This is generally believed to result from increased energy expenditure in peripheral tissues, but some hyperthyroid patients increase their food intake enough to gain weight (Gurney et al., 1970), suggesting that it is not simply a compensatory change. TH signalling may be important to initiate the drive to feed as deiodinase 2, the enzyme that activates TH signalling by converting thyroxine (T4) into the transcriptionally more active triiodothyronine (T3), is upregulated by fasting in the hypothalamus (Diano et al., 1998), the region of the brain controlling feeding, growth, and reproductive status. Moreover, acute and chronic administration of low doses of T3 has been shown to increase feeding without affecting energy expenditure (Kong et al., 2004). These observations suggest that TH has central, as well as peripheral effects on the regulation of feeding behavior and metabolic states. Hyperthyroid rats show an upregulation of the orexigenic gene *agouti‐related protein* (*Agrp*) in the hypothalamus accompanied by a decrease in *proopiomelanocortin* (*Pomc*), the precursor of the anorexigen alpha‐melanocyte‐stimulating hormone (α‐MSH; Varela et al., 2012). In addition, the importance of TH signalling in the hypothalamus is highlighted in the regulation of energy balance of seasonal animals (Barrett et al., 2007). This highly conserved pathway is now considered to be the basis of long‐term changes in energy balance, growth and reproduction, in birds and mammals. In Siberian hamsters, hypothalamic implants releasing T3 promote long day‐like (i.e. summer‐like) reproductive and body weight responses (Murphy et al., 2012) and conversely local delivery of T3 is able to block short day (winter‐like)‐induced weight loss (Barrett et al., 2007). From these findings it is now generally concluded that local hypothalamic T3 availability is responsible for long‐term seasonal changes in energy metabolism.

The observations described above suggest that TH signalling is an important regulator of hypothalamic function. TH is thought to control genes affecting growth and energy balance in seasonal mammals including *Agrp*, *Ghrh*, and *Pomc* (Ross et al., 2009), although this control may be indirect. However despite considerable investigation, the genes directly regulated by TH in the hypothalamus are largely unknown; indeed, the genes regulated by TH in much of the brain remain undiscovered. Retinaldehyde dehydrogenase 1 (Raldh1, also known as Aldh1a1) is regulated by a number of hormone activators of nuclear receptors, such as estrogen (Fujiwara et al., 2009) and androgen (MacLean et al., 2008), as well as metabolite‐regulated nuclear receptors such as LXR (Huq et al., 2006). Via its synthesis of retinoic acid (RA) from retinaldehyde, Raldh1 itself then controls the activity of further nuclear receptors, the retinoic acid receptors (RARs). In the hypothalamus, Raldh1 is specifically localized to tanycytes, radial glia‐like cells lining the third ventricle, potentially acting to communicate between the cerebrospinal fluid, circulation, and hypothalamic neurons (Shearer et al., 2010). They have an intermediary role to play in the control of appetite and energy balance (Bolborea and Dale, 2013) and help to mediate molecular exchange between blood, brain, and cerebrospinal fluid (Langlet et al., 2013), including transport of leptin (Balland et al., 2014). *Raldh1* was explored *in vivo* as a TH‐regulated gene in the rat hypothalamus and was found to be rapidly induced and potentially under direct TH control. Gene regulation by RA was studied in an organotypic slice culture system developed for this study. This system was free of secondary *in vivo* influences but maintained the structures necessary for hypothalamic function. It was shown that RA regulates the expression of hypothalamic genes known to affect energy balance and growth and may act as an intermediary in the action of TH in the hypothalamus. Further, it was demonstrated that *Rarb* and *Ghrh* were epigenetically repressed and the RA signalling pathway may be a means of epigenetic control of gene expression in the hypothalamus.

## Materials and Methods

### Animals

Sprague Dawley rats were bred in the University of Aberdeen animal facility. Fischer F344/N male rats were supplied by Harlan Sprague Dawley Inc. (Indianapolis, USA). All animals were kept in a 12h:12h light:dark cycle with unlimited access to food and water. All procedures conformed to Home Office regulations and local ethics committee guidelines.

### T3 Administration

T3 (Sigma Aldrich) was dissolved in 1 N NaOH at 1 mg/ml and then diluted to 40 µM in phosphate‐buffered saline (PBS), pH 7.4. Eight‐week‐old male Sprague Dawley rats were injected subcutaneously with 65 µg/kg (100 nmol/kg) T3. Control animals were injected with an equivalent volume of 1 N NaOH diluted in PBS. 4 hours post‐injection, the animals were killed and the hypothalami were dissected and rapidly frozen on dry ice.

### Nissl Staining

Male rat pups were transcardially perfused with saline followed by 4% paraformaldehyde (PFA) in phosphate buffer. The brains were removed and incubated overnight at 4°C in 4% PFA, washed in PBS and transferred to 30% sucrose in PBS. 40 µm‐thick coronal brain sections were cut using a cryostat, mounted on polylysine‐coated slides, and allowed to dry. Tissue sections were stained with cresyl violet, dehydrated through an ethanol series into xylene, and mounted with DPX (Fisher Scientific).

### Hypothalamic Organotypic Slice Cultures

To exclude circadian‐driven changes in gene expression, all hypothalamic slice cultures were set up at the same time of day (commencing at ZT07‐8). P10‐12 male rat pups were euthanized with Euthatal. The brains were removed under sterile conditions and placed in ice‐cold slice culture medium consisting of 50% minimal essential medium, 25% Hank's buffered salt solution, 25% heat‐inactivated horse serum, containing penicillin‐streptomycin and Glutamax, supplemented with 5 mg/ml additional glucose and buffered with 25 mM HEPES. The cortices were removed and the brains were sliced into 400 µm coronal sections using a McIlwain tissue chopper. Slices were transferred into cold medium, separated using forceps under a dissection microscope and slices containing the third ventricle were isolated. Each hypothalamus yielded five to six slices. The slices were trimmed and cut in half along the midline, giving two sets of slices per animal, with each containing the same (but alternate) regions. Each sample consisted of one set of slices. After incubation in ice‐cold culture medium for 1 to 2 hours, each set of slices was transferred onto a Millicell‐CM cell culture insert (Millipore) in a six‐well plate using a sterile glass pipette. Excess medium was removed from the tissue and 1 ml of fresh slice culture medium was added below the insert. Slices were transferred to 35°C, 5% CO_2_. After 24 hours, the medium was removed and replaced with serum‐free, vitamin A‐deficient medium consisting of Neurobasal medium containing B27 supplement without vitamin A, penicillin‐streptomycin and Glutamax (all reagents from Invitrogen) and 5 mg/ml additional glucose. Slices were incubated at 35°C, 5% CO_2_ for a further 3 days. Slices were maintained in vitamin A‐deficient medium to deplete the tissue of retinol and minimize retinoic acid synthesis by endogenous Raldh1.

The medium was replaced with fresh serum‐free, vitamin A‐free medium and slices were treated with 10 mIU bovine thyroid‐stimulating hormone (TSH), 50 nM T3, 10 nM, or 1 µM all‐*trans*‐RA (all from Sigma Aldrich), 50 ng/ml trichostatin A (Cayman Chemical Co.) or 100 µM sirtinol (Tocris). Sirtinol, retinol and RA were dissolved in DMSO and therefore an equivalent volume of DMSO (0.1%) was added to wells containing control slices. One set of slices from each animal was treated with the other set being used as control. Treatment times are indicated in figure legends. After treatment, slices were fixed in 4% paraformaldehyde for 2 hours at room temperature for immunohistochemistry or excised from the culture inserts, transferred to microcentrifuge tubes and frozen rapidly on dry ice for RNA extraction.

### Slice Immunohistochemistry

After 6 days *ex vivo*, hypothalamic slices were washed with PBS and fixed by immersion in 4% PFA for 2 hours at room temperature. Slices were labeled using antibodies against vimentin (V9, Sigma Aldrich) and Darpp‐32 (19A3, Cell Signaling) and appropriate fluorescent secondary antibodies (Jackson ImmunoResearch). The membranes carrying the slices were transferred onto microscope slides and mounted using mounting medium containing Hoechst. Labelling was visualized by fluorescence microscopy.

### Primary Tanycyte Culture

Primary tanycyte cultures were prepared from 10‐day‐old male Sprague Dawley rat pups as previously described (Bolborea et al., 2015; De Seranno et al., 2004; Prevot et al., 2003). Brains were removed under sterile conditions, placed in ice‐cold DMEM/F‐12 medium containing penicillin/streptomycin and 25 mM HEPES and the median eminence dissected. Median eminences from 8–10 pups were pooled together, dissociated and plated out in DMEM/F‐12 containing 10% foetal calf serum and penicillin/streptomycin in a 25 cm^2^ cell culture flask. Medium was replaced every 3 days. The composition of primary tanycyte cultures was assessed after 8 to 10 days *in vitro* by immunohistochemistry for the tanycyte marker vimentin and the astrocyte marker GFAP; all cells expressed vimentin, but only a small number expressed GFAP. RNA was also extracted from cultured cells and tested by PCR for expression of the tanycyte markers *Dio2*, *Darpp32*, *Rax*, *Tshr*, and *Gpr50*.

Primary cultured tanycytes were transferred to 12‐well plates for experiments after 8‐10 days *in vitro*. The day after plating out, tanycytes were treated with 50 nM T3 or vehicle for 24 hours. RNA was extracted from treated cells for qPCR analysis.

### Quantitative Polymerase Chain Reaction

Total RNA was extracted from slices using a Qiagen RNeasy RNA purification kit. cDNA was synthesized from 500 ng total RNA using High Capacity RNA‐to‐cDNA Master Mix (Applied Biosystems Ltd). *Dio2* and *Dio3* primers were obtained from Qiagen (QuantiTect Primer Assays Rn_Dio2_2_SG and Rn_Dio3_1_SG, respectively); other primers (Table 1) were designed using PrimerBLAST (Ye et al., 2012). qPCR reactions were set up using SensiMix SYBR master mix (Bioline) and were run on a Roche LightCycler 480 and analysed using LightCycler 480 1.5 software. Expression of genes of interest was normalized to *Actb* levels. Standard curves and blank controls were run for all sets of primers. Results shown are from a minimum of two independent experiments per condition. Gene expression in T3‐injected rats was compared to that of vehicle‐injected controls using unpaired Student's t‐tests. Expression in treated hypothalamic slices was compared to control slices from the same individuals by paired Student's t‐tests or ANOVA.

**Table 1:** Sequences of primers used for RT‐PCR and qPCR

Gene	RefSeq code	Product size (bp)	Primer sequences	
			Forward	Reverse
*Actb*	NM_031144.3	112	CCACACCCGCCACCAGTTCG	GACGGCCCGGGGAGCATCGT
*Agrp*	NM_033650.1	149	GGGCGTGGCACCACTGAAGG	GTGGATCTAGCACCTCTGCCAAAGC
*Cart*	NM_017110.1	83	AAGTCCCCATGTGTGACGCTGGA	TCCTCGGGGACAGTCACACAGC
*Cyp26b1*	NM_181087.2	127	TCCATTGGCGACATCCACCGC	GGCTGCTCCAGGCTCGAAGTG
*Darpp32*	NM_138521.1	127	ATGGACCCCAAGGACCGCAAGAA	CTGAGACCCGGAACAGCAAGGC
*Ghrh*	NM_031577.1	147	GGCAGCAAGGGGAGAGGAACCA	CGAGGGCTCAAGCCTCCGC
*Gpr50*	NM_001191915.1	147	GCGCAATGGTCATCACTGTCGTC	ACGGGTAGATGGCCACGAGCA
*Npy*	NM_012614.1	142	GCCAGATACTACTCCGCTCTGCGA	CTTCAAGCCTTGTTCTGGGGGCA
*Pcsk2*	NM_012746.1	222	CGTGGGGGCAAAGGCAGCAT	TGTGGTAGCCACACCGGCCT
*Pomc*	NM_139326.2	225	TGCCTTTCCGCGACAGAGCC	TGCCTGGAAACACGGGCGTC
*Raldh1*	NM_022407.3	196	ACGTGGAAGAAGGGGACAAGGCTG	GCAAAGACTTTCCCACCATTGAGTGCC
*Rarb*	NM_031529.1	134	ACACCACGAATTCCAGCGCTGAC	CAGACCTGTGAAGCCCGGCA
*Rax*	NM_053678.1	98	CGACGTGTACAGCCGCGAAGA	GGCGTCTCCACTTGGCTCGAC
*Tshr*	NM_012888.1	118	GGGTGTACTTCTCCACCCTGCGA	TCTCGATGAGCTTCAGAGTCTGGGTG

## Results

### Peripheral Administration of T3 Upregulates Hypothalamic Raldh1 Expression

Thyroid hormone signalling in the hypothalamus is thought to play a crucial role in the control of energy balance, but little is known about its direct targets in the hypothalamus. To investigate potential targets of TH signalling in the hypothalamus, male Sprague Dawley rats were given a single subcutaneous injection of 100 nmol/kg T3 at 8 weeks of age. Treated rats were killed 4 hours post‐injection and the hypothalami were rapidly removed for qPCR analysis of hypothalamic genes known to affect energy balance.

**Figure 1:**
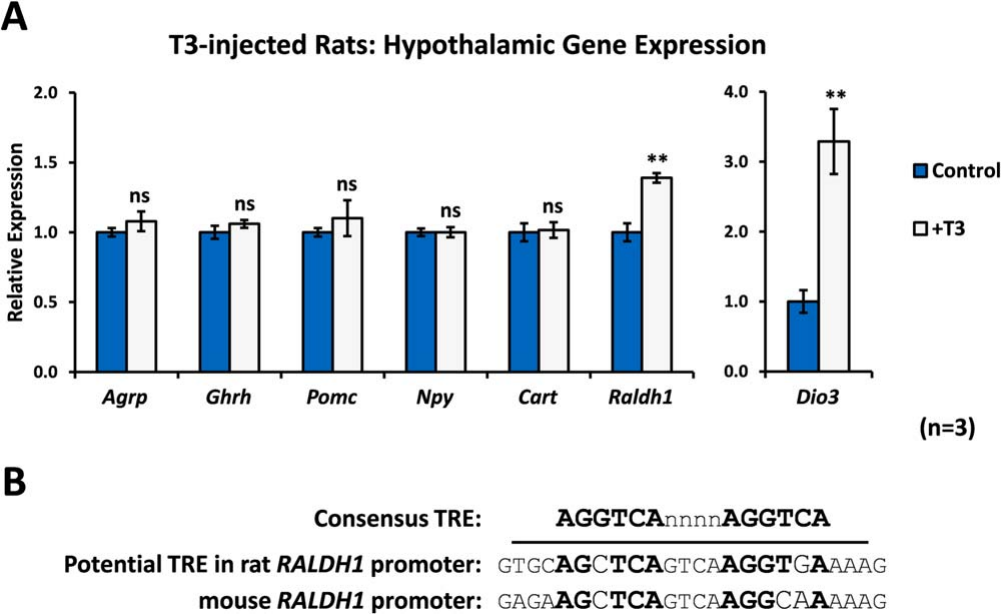
T3 upregulates *Raldh1* expression in the rat hypothalamus *in vivo*. **A**: Eight‐week old male Sprague Dawley rats were injected subcutaneously with 100 nmol/kg T3. The hypothalamus was removed 4 hours after T3 injection for qPCR analysis. Expression of *Agrp*, *Pomc*, *Npy*, *Cart*, and *Ghrh* was unaffected by short‐term T3 treatment, but hypothalamic *Raldh1* was significantly upregulated 4 hours after T3 injection, compared with vehicle‐injected animals. *Dio3* was used as a positive control for the activity of T3. *N* = 3 animals per treatment group. **B**: A sequence closely matching the consensus sequence of a DR4‐type thyroid hormone response element (TRE) was identified in the rat *Raldh1* promoter, suggesting direct regulation of *Raldh1* expression by T3. Statistical significance was assessed using Student's *t*‐test. ** *P* < 0.01. [Color figure can be viewed in the online issue, which is available at wileyonlinelibrary.com.]

*Deiodinase 3* (*Dio3*) has previously been shown to be directly induced by T3 (Barca‐Mayo et al., 2011; Bianco et al., 2002) and was used as a positive control. *Dio3* was strongly upregulated in the hypothalamus of T3‐treated rats (Fig. 1A), confirming that peripheral administration of T3 increased hypothalamic T3 and that the time between administration and dissection was sufficient for alterations in hypothalamic gene expression. Despite the presence of T3 in the hypothalamus, no significant changes in the expression of *agouti‐related protein* (*Agrp*), *growth hormone‐releasing hormone* (*Ghrh*), *neuropeptide Y* (*Npy*), *cocaine‐ and amphetamine‐regulated transcript* (*Cart*) or *proopiomelanocortin* (*Pomc*) were observed in T3‐treated animals after 4 hours (Fig. 1A).

*Raldh1*, which encodes the RA synthetic enzyme retinaldehyde dehydrogenase 1 present in tanycytes (Shearer et al., 2010), was investigated as a gene regulated by several nuclear receptor family members (Fujiwara et al., 2009; Huq et al., 2006; MacLean et al., 2008). This gene was significantly upregulated in the hypothalamus of T3‐treated rats (Fig. 1A). In addition, a sequence with high similarity to a DR4‐type TH response element (TRE; AGCTCAgtcaAGGTGA; Fig. 1B) was identified in the promoter of the rat *Raldh1* gene, close to the transcription start site. The upregulation of *Raldh1* just 4 hours after T3 administration and the presence of a potential TRE in the promoter of the rat *Raldh1* gene suggest that *Raldh1*, and so RA synthesis, may be a direct target of TH signalling in the hypothalamus.

### Cultured Hypothalamic Slices with Tanycytes Are Responsive to Thyroid Stimulating Hormone

**Figure 2:**
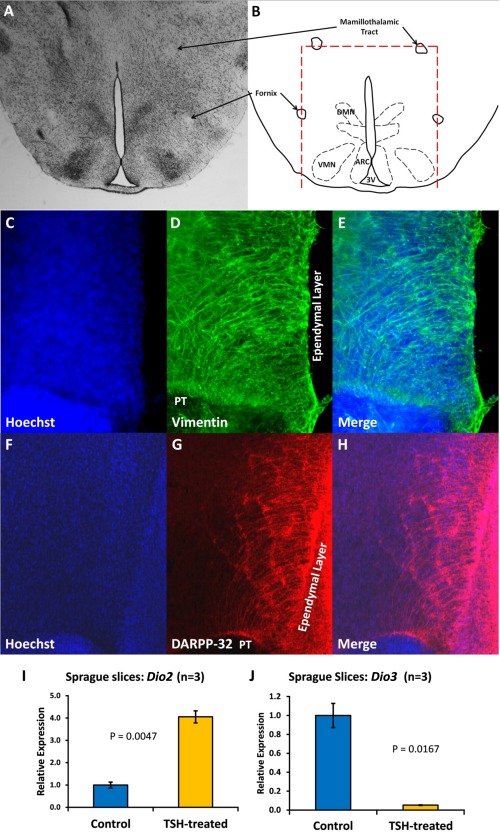
Hormone‐responsive tanycytes are maintained in hypothalamic organotypic culture. 400 μm coronal slices were cut through the hypothalamus of P10‐12 Sprague Dawley rat pups. The mammillothalamic tract and fornix (**A, B**) were used as anatomical landmarks to trim the slices. Nissl staining of P10 rat sections (**A**) shows the location of the DMN, VMN and arcuate (ARC) nuclei in relation to these landmarks. Slices fixed after 6 days of culture in vitamin A‐deficient medium and labelled using antibodies against the tanycyte markers vimentin (**C–E**) and dopamine‐ and cAMP‐regulated phosphoprotein (DARPP‐32; **F–H**) show that tanycytes are present in cultured slices. To test the response of tanycytes in cultured hypothalamus to hormonal signals, slices were treated with 10 mIU thyroid‐stimulating hormone (TSH) for 48 hours before qPCR analysis of gene expression. TSH upregulated *Dio2* (I) and downregulated *Dio3* (**J**), demonstrating that tanycytes are not only present after 6 days *ex vivo*, but respond as expected to TSH in terms of gene expression. [Color figure can be viewed in the online issue, which is available at wileyonlinelibrary.com.]

The function of T3 and RA in the hypothalamus was further explored using an *ex vivo* organotypic culture system. This technique was modified from that of House et al. (1998) and allowed T3 and RA to be studied independently of feedback from the rest of brain or body. The brain was removed from P10‐12 male Sprague Dawley rat pups and cut into 400 µm‐thick coronal slices. Slices were trimmed dorsally at the level of the mammillothalamic tract and laterally at the fornix, both of which were visible in the slices (Fig. 2A, B). These slices contained the ventricular region and median eminence (ME) in addition to the arcuate (ARC), ventromedial (VMN), dorsomedial (DMN), and paraventricular (PVN) hypothalamic nuclei. Trimming the slices using visible anatomical cues minimized variation but allowed for differences in brain size between individual animals. The slices were standardly cultured in serum‐free vitamin A‐free medium for 72 hours before treatment, depleting the tissue of vitamin A and thereby preventing endogenous synthesis of retinoic acid.

Crucially for these studies, these slices could be cultured in a way that maintained health of the neurons, the integrity of the hypothalamic nuclei and also the viability of cells vital to TH and RA signalling, the tanycytes. The tanycytes are specialized radial glia‐like cells in the cell layer lining the third ventricle thought to be critical for the transduction of extrahypothalamic signals into the parenchyma. Tanycytes are the cells in which both RA is synthesized (Shearer et al., 2010; Shearer et al., 2012b) and thyroxine (T4) is converted to the more active T3 (Yasuo et al., 2007). To confirm the presence of tanycytes in cultured rat hypothalami, slices were maintained *ex vivo* for 6 days and then fixed in 4% PFA. Immunohistochemistry using antibodies against the tanycyte markers vimentin (Fig. 2C‐E; Leonhardt et al., 1987) and dopamine‐ and cAMP‐regulated phosphoprotein (DARPP‐32, Fig. 2F‐H; Meister et al., 1988) demonstrated that tanycytes were present after 6 days of *ex vivo* culture, with processes projecting into the parenchyma of the hypothalamus.

Short‐term slice cultures of adult mouse hypothalamus have been shown to be responsive to thyroid‐stimulating hormone (TSH) after 4 hours *ex vivo*, upregulating *Dio2* expression and downregulating *Dio3* in the ependymal cell layer of the ventromedial hypothalamus (Unfried et al., 2009). TSH was used as a positive control to test the response of tanycytes in cultured rat slices to a hormonal signal. Slices were maintained in culture for 4 days, then treated for 48 hours with 10 mIU bovine TSH before qPCR analysis. TSH treatment of cultured slices induced a 4‐fold increase in *Dio2* expression (P=0.005; Fig. 2I) and reduced *Dio3* by 95% (*P* = 0.02; (Fig. 2J). These data demonstrate that tanycytes are maintained in cultured rat hypothalamus for up to 6 days and that cultured tissue can behave like the *in vivo* hypothalamus in terms of hormonal control of gene expression.

### T3 Upregulates Raldh1 Expression in Cultured Hypothalamus

A key function for TSH in the hypothalamus is to increase deiodinase 2 and decrease deiodinase 3 in cells, including tanycytes, of the ependymal layer of the hypothalamus (Helfer et al., 2013) and so locally increase T3 levels, the most transcriptionally active form of TH. T3 is known to signal within the hypothalamus and is essential as a mediator for environmental regulators of growth and energy balance (Mullur et al., 2014). However, the genes on which T3 directly acts upon to bring about these changes in the hypothalamus are poorly understood. Given *Raldh1*'s induction by T3 *in vivo* (Fig. 1A), this was tested in the *ex vivo* slice culture assay. Slices were treated with 50 nM T3 for 48 hours before analysis of gene expression by qPCR.

**Figure 3:**
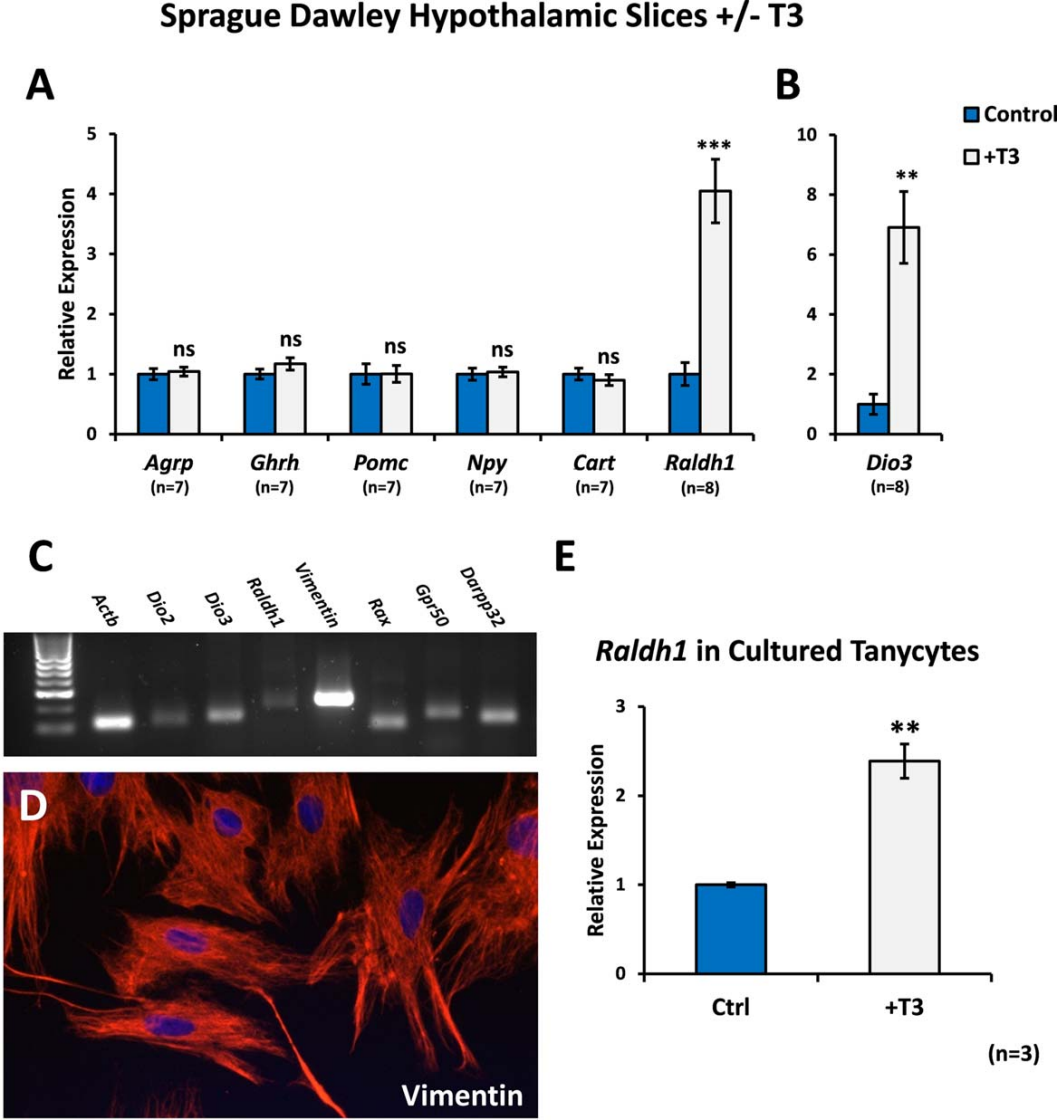
T3 upregulates *Raldh1* in organotypic cultures of rat hypothalamus. **A**: Hypothalamic slices from P10 male Sprague Dawley rats were cultured for 4 days and then treated for 48 hours with 50 nM T3 before qPCR analysis. As *in vivo*, *Raldh1* was upregulated in T3‐treated hypothalamic slices. *Agrp*, *Ghrh*, *Pomc*, *Npy* and *Cart* were unaffected by T3. **B**: *Dio3* was used as a positive control for T3 activity. Numbers of samples are shown. **C**: Primary cultures of tanycytes expressed tanycyte markers *Dio3, Vim, Rax, Gpr50 and Darpp‐32*. Some markers such as *Dio2*, were only weakly expressed and this was also the case for *Raldh1*. **D**: The cultured tanycytes strongly expressed vimentin by immunohistochemistry and several of the cells extended long processes. **E**: Addition of 50nM of T3 to cultured tanycytes significantly induced their expression of *Raldh1*. Statistical significance was assessed using paired Student's *t*‐test. ** *P* < 0.01; *** *P* < 0.001. [Color figure can be viewed in the online issue, which is available at wileyonlinelibrary.com.]

*Raldh1* was potently induced by T3, with a 4‐fold increase in expression in T3‐treated hypothalamic slices (*P* < 0.001; Fig. 3A), indicating that activation of thyroid hormone signalling in the hypothalamus itself is sufficient to upregulate *Raldh1* expression and therefore regulate synthesis of RA in the hypothalamus. Thus, regulation of *Raldh1* expression, and therefore potentially the rate of RA synthesis, in the hypothalamus may provide one route by which T3 can bring about changes in hypothalamic gene expression.

Induction of *Raldh1* was similar to that of the T3 catabolic enzyme *Dio3*, previously shown to be upregulated by T3 (Bianco et al., 2002) and with a TRE in its promoter (Barca‐Mayo et al., 2011) and which was increased over six‐fold in T3‐treated slices compared to controls (Fig. 3B; *P* = 0.0003). Both the rat and mouse *Raldh1* promoters contain a potential TRE (Fig. 1B). In contrast, T3 did not significantly alter expression of *Agrp*, *Ghrh* or *Pomc*, genes thought to be regulated by TH *in vivo* in F344 rats (Ross et al., 2009) as part of its action to control weight and energy balance (Fig. 3A). Similarly, other genes known to be involved in hypothalamic regulation of these processes, *Npy* and *Cart*, were unaffected by T3 (Fig. 3A).

*In vivo*, the only cells of the hypothalamus that normally express Raldh1 are the tanycytes (Shearer et al., 2012b), the same cells in which T4 is converted to T3 by deiodinase 2 (Dio2). To confirm whether *Raldh1* can be induced in tanycytes by T3, primary tanycyte cultures were established following previously described protocols (Bolborea et al., 2015; De Seranno et al., 2004; Prevot et al., 2003) which are approximately 95% pure. These cells expressed the typical markers of tanycytes including *Dio3, Vim, Rax, Gpr50 and Darpp‐32* (and weakly *Dio2* and *Raldh1*) by PCR (Fig 3C) and vimentin by immunochemistry (Fig 3D) and T3 was found to significantly induce *Raldh1* by 2.5 fold (*P* = 0.0020; Fig. 3E).

**Figure 4:**
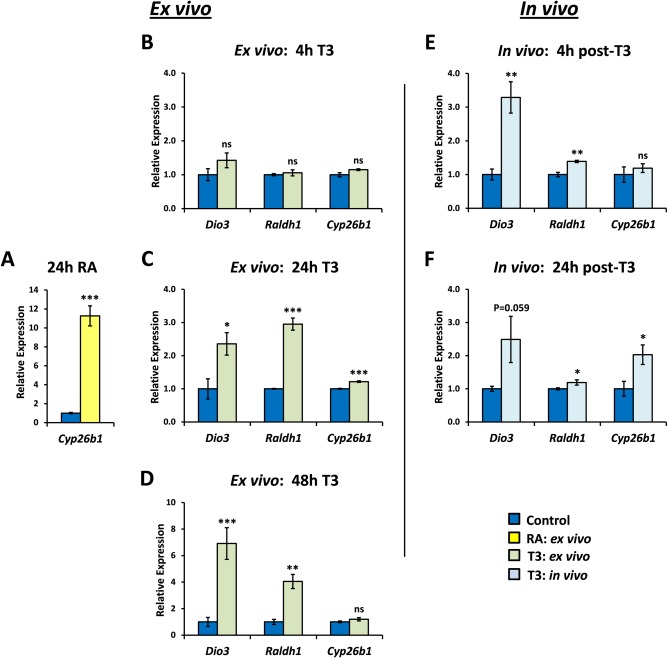
Time course of *ex vivo* and *in vivo* induction of genes in the hypothalamus by thyroid hormone. The relative timing of gene induction by T3 was examined for *Dio3*, as a positive control *Raldh1*, the RA synthetic enzyme of the hypothalamus and *Cyp26b1*, a RA‐responsive gene. *Cyp26b1* was demonstrated to respond to RA by direct addition of RA to hypothalamic slices (**A**). None of the three genes examined were responsive within 4 hours of addition of T3 to hypothalamic slices (**B**) but significant increases of both *Dio3* and *Raldh1* were evident after 24 (**C**) or 48 hours T3 treatment (**D**). *Cyp26b1* was only weakly (but significantly) induced at 24 hours (C). *In vivo*, *Dio3* and *Raldh1* were both significantly and rapidly induced by 4 hours (**E**) and, at least for *Raldh1*, maintained for 24 hours (**F**). *Cyp26b1* did not respond as rapidly *in vivo* but was induced two‐fold by 24 hours (F) and thus follows the expression of the *Raldh1* gene necessary for RA synthesis. Statistical significance was assessed using unpaired Student's t‐test. * *P* < 0.05; ** *P* < 0.01; *** *P* < 0.001. [Color figure can be viewed in the online issue, which is available at wileyonlinelibrary.com.]

To investigate whether RA may be synthesized locally in the hypothalamus by TH‐induced Raldh1 the expression of a gene regulated by RA, and not by TH in the hypothalamus, was investigated. *Cyp26b1* is highly RA‐inducible in hypothalamic slices (*P* = 0.0006; Fig. 4A) but was not induced by 50 nM T3 after 4 or 48 hours (Fig. 4B, D). Weak, although significant induction is seen after incubation with T3 for 24 hours (Fig. 4C) which may represent a weak response to T3 or possibly may result from low amounts of RA generated from endogenous retinol in the slices, mediated by T3‐induced Raldh1. In contrast to *Cyp26b1*, treatment of hypothalamic slices with T3 resulted in robust induction of both *Dio3* (as a positive control; *P* = 0.025) and *Raldh1* (*P* < 0.0001) in hypothalamic slices after 24 hours (Fig. 4C) which was maintained at 48 hours (Fig. 4D).

The influence of TH on the same set of genes was then investigated *in vivo*. TH rapidly induced both *Dio3* and *Raldh1* (*P* = 0.0097 and *P* = 0.006, respectively; Fig. 4E). This was faster *in vivo* than ex *vivo*, suggesting that culture of slices possibly results in a decline in the speed of tissue responsiveness or that *in vivo* TH is perhaps transported or concentrated more effectively in the hypothalamus, or possibly that extrahypothalamic effects on tanycytes may potentiate *Raldh1* expression *in vivo*. *Cyp26b1* was not induced so rapidly by TH (Fig. 4E) but significant 2‐fold induction was evident by 24 hours (*P* = 0.0192; Fig. 4F) following sequentially from the early increase in *Raldh1*. This temporal delay in *Cyp26b1* induction would be expected if *Cyp26b1* was responding not to exogenous T3, but to an increase in RA synthesis by Raldh1.

### RA Upregulates Growth‐Associated Genes in Cultured Hypothalamus

**Figure 5:**
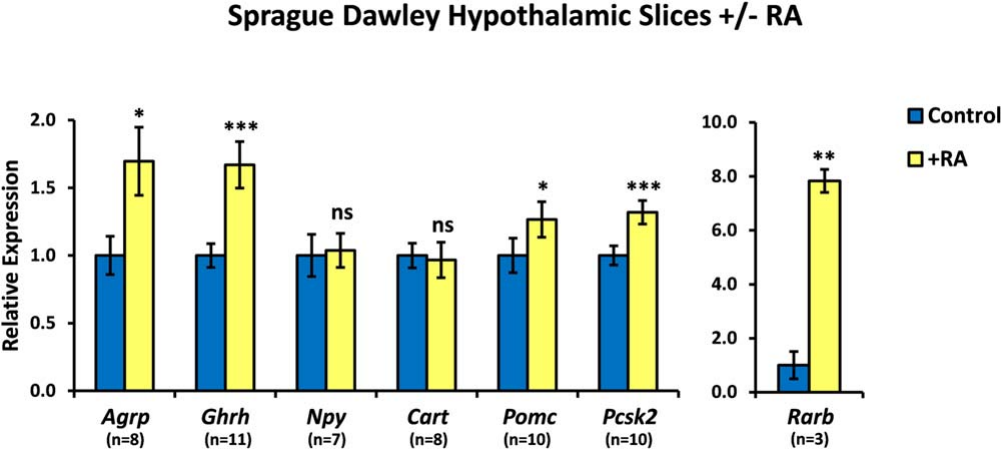
Retinoic acid regulates expression of *Agrp* and *Ghrh* in the rat hypothalamus. Hypothalamic slices from male Sprague Dawley rats were cultured for 4 days and then treated for 48 hours with 1 μM RA before qPCR analysis. *Agrp* and *Ghrh* expression was significantly upregulated in RA‐treated slices. *Pomc* and *Pcsk2* showed smaller, but still significant increases in expression in RA‐treated cultures. *Npy* and *Cart* were unaffected by RA treatment. *Rarb* was used as a positive control for RA activity. Numbers of samples are shown. Statistical significance was assessed using paired Student's *t*‐test. * *P* < 0.05; ** *P* < 0.01; *** *P* < 0.001. [Color figure can be viewed in the online issue, which is available at wileyonlinelibrary.com.]

If RA may act as a downstream mediator of TH's actions within the hypothalamus then it would be presumed that RA may induce some of the growth‐associated genes which TH does not directly control. Hypothalamic slice cultures were treated with 1 µM RA for 48 hours before qPCR analysis. *Rarb* was used as a positive control for RA activity, as its promoter contains a well‐characterized RA response element (RARE; Leid et al., 1992). *Rarb* expression was 7.8‐fold higher in RA‐treated slices than controls (*P* = 0.004; Fig. 5).

In cultured slices, RA significantly upregulated expression of the orexigenic gene *Agrp* (70% increase relative to control; *P* = 0.012; Fig. 5) as well as *Ghrh* (67% increase; *P* < 0.001). *Npy* and *Cart* expression was unaffected by RA treatment (Fig. 5). *Pomc* showed a small but significant increase in expression in RA‐treated slices (27% increase; *P* = 0.040). The gene encoding PC2, a prohormone convertase involved in the processing of POMC into hormones including ACTH and α‐MSH (Pritchard et al., 2002), was also upregulated by RA treatment (*Pcsk2*; 32% increase; *P* < 0.001).

**Figure 6:**
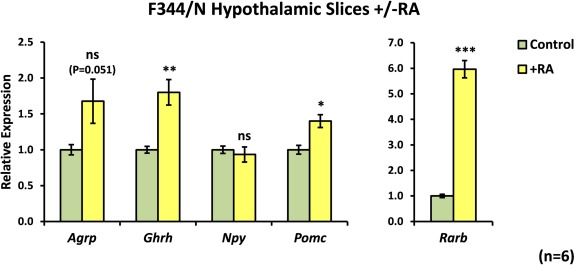
The F344/N rat hypothalamus is also responsive to RA. Hypothalamic slices from male F344/N rats were cultured for 4 days and then treated for 48 hours with 1 μM RA before qPCR analysis. Responses to RA in slices from F344/N rat pups were very similar to those in Sprague Dawley slices. *Ghrh* was significantly upregulated in RA‐treated slices, with smaller but significant increases in *Pomc*. *Agrp* appeared upregulated to a similar extent to that observed in Sprague Dawley slices (Fig. 4), although this did not reach statistical significance in this case. *Rarb* was used as a positive control for the activity of RA. *N* = 6 per group for all genes. Statistical significance was assessed using paired Student's *t*‐test. * *P* < 0.05; ** *P* < 0.01; *** *P* < 0.001. [Color figure can be viewed in the online issue, which is available at wileyonlinelibrary.com.]

The experiments described thus far were performed in hypothalamic slices from outbred Sprague Dawley rats. To determine if the effects of RA are applicable to multiple strains of rat, hypothalamic slice cultures were established using tissue from another commonly used rat strain, the photosensitive inbred Fischer F344/N. Slices from F344/N rat pups were treated with 1 µM RA after 4 days *ex vivo* and harvested after 48 hours for qPCR analysis (Fig. 6). The response of F344/N hypothalamic slices to RA was similar to that of the Sprague Dawley hypothalamus (Fig. 6). As was observed in Sprague Dawley slices, RA induced significant upregulation of *Rarb* (5.96‐fold, *P* < 0.001) and *Ghrh* expression (1.80‐fold, *P* = 0.004) in the F344/N hypothalamus. Smaller but significant increases in the expression of *Pomc* (1.40‐fold, *P* = 0.022) were seen with RA. RA treatment of F344/N hypothalamus increased *Agrp* expression by a similar amount to that seen in Sprague Dawley slices (F344/N: 1.68‐fold increase, *P* = 0.051; Sprague Dawley: 1.70‐fold increase, *P* = 0.012), although this increase was not quite significant. *Npy* expression was unaffected by RA. Together, these data suggest that the hypothalamic response to RA treatment is similar between the two rat strains.

### Hypothalamic RA Signalling Is under Epigenetic Control

Epigenetic regulation of gene expression via DNA/histone modifications is known to control aspects of hypothalamic function, such as hormone synthesis (Miller et al., 2011) and sexual maturation of the brain (Matsuda et al., 2011). In some cases, this is believed to be a behavioural control mechanism that, for instance, contributes to imprinting action of the environment on the young animal that results in altered behaviour in the mature animal. Epigenetic control via histone acetylation is particularly active in promoting RA signalling, and derepression of RA signalling may be a major rate‐limiting target of histone deacetylase (HDAC) inhibitors (Epping et al., 2007). To explore whether the RA signalling system in the hypothalamus is acted upon by such an epigenetic mechanism, cultured hypothalamic slices were treated with a low concentration of RA (10 nM) which results in weaker induction of gene expression. This was combined with trichostatin A (TSA), a class I/II HDAC inhibitor (HDACI), or sirtinol, an inhibitor of the sirtuin (class III) family of HDACs.

**Figure 7:**
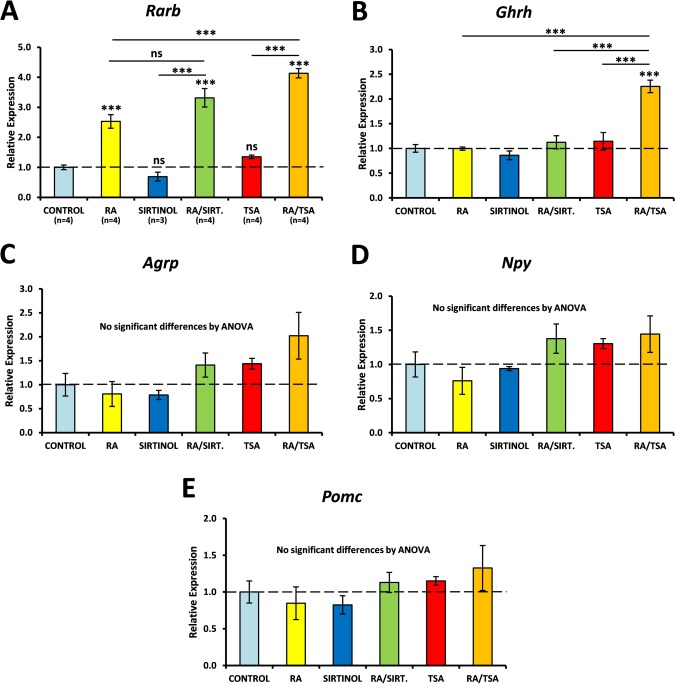
RA regulation of gene expression in the hypothalamus is subject to epigenetic control by class I/II histone deacetylases. After 4 days *ex vivo*, F344/N hypothalamic slices were treated for 48 hours with trichostatin A (TSA), a class I/II histone deacetylase inhibitor (HDACI), or sirtinol, a class III HDACI, in the presence or absence of 10 nM RA. qPCR analysis was performed using primers for *Rarb*, *Ghrh*, *Agrp*, *Npy* and *Pomc*. *Rarb* was upregulated by 10 nM RA and TSA, but not sirtinol, enhanced the effect of RA (**A**). *Ghrh* expression was significantly upregulated by 10 nM RA only in the presence of TSA (**B**). *Agrp*, *Npy* and *Pomc* were unaffected by HDACIs (**D–E**). Numbers of samples per condition are shown in (A) and were the same for each gene examined. Data were analysed by ANOVA followed by Tukey's *post hoc* tests. ** *P* < 0.01; *** *P* < 0.001. [Color figure can be viewed in the online issue, which is available at wileyonlinelibrary.com.]

In cultured slices from F344/N rats, 10 nM RA was sufficient to increase expression of *Rarb* (2.5‐fold increase relative to controls; Fig. 7A), but not *Ghrh* (Fig. 7B). TSA alone did not affect *Rarb* expression, but potentiated the response to RA (4.1‐fold increase relative to control; 64% higher than RA alone). *Ghrh* expression was not altered by 10 nM RA or TSA alone, but RA and TSA in combination induced a 2.25‐fold increase in *Ghrh* (Fig. 7B). Sirtinol had no effect on *Ghrh* expression and although the response of *Rarb* to RA was higher in the presence of sirtinol (3.3‐fold increase relative to controls) than with RA alone (2.5‐fold; Fig. 7A), the difference was not significant. Expression of *Agrp*, *Pomc* and *Npy* was not significantly altered by 10 nM RA even in the presence of TSA or sirtinol (Fig. 7C‐E). These data suggest that the expression of some RA‐responsive genes in the hypothalamus may be further regulated as a result of epigenetic chromatin modifications by class I/II HDACs.

## Discussion

TH controls growth‐associated physiology but T3, the most active TH metabolite, does not directly regulate genes involved in this process and gene targets of TH in the hypothalamus have proved enigmatic (Barrett et al., 2007). This study shows that, *in vivo*, T3 very rapidly induces expression of a key enzyme, *Raldh1*, required for RA synthesis by tanycytes, the source of RA for the hypothalamus (Shearer et al., 2010). That RA levels rise in the hypothalamus on addition of TH is suggested by the induction of a RA reporter gene, *Cyp26b1*, a gene relatively refractory to TH. An organotypic culture system was then developed as a simple system to identify genes immediately downstream of RA and demonstrated that RA (but not T3) has the potential to induce several growth‐associated genes. Thus, the capacity of TH to increase RA synthesis provides a putative mechanism by which TH may control RA‐inducible genes which may include those that regulate appetite and growth. Further, it was shown that the RA signalling system in the hypothalamus is itself partially repressed by an epigenetic mechanism (histone deacetylation) and can be stimulated by inhibition of histone deacetylases.

RA has only recently been recognized to be active in the hypothalamus as a regulatory factor and the hypothalamus is one of the few brain regions in which RA functions, alongside regions such as the hippocampus and olfactory system (Goodman et al., 2012; Hagglund et al., 2006; Shearer et al., 2012a). However the importance of RA and its precursor, vitamin A, to control feeding and weight, was recognized much earlier. The sudden removal of vitamin A from the diet of rats causes rapid weight loss and a reduction in food intake which can be countered by administration of RA (Anzano et al., 1979). Weight loss induced by vitamin A deficiency (VAD) persists even in force‐fed animals, indicating that VAD affects the fundamental mechanisms of weight control in addition to regulating feeding behaviour. Further evidence for the involvement of vitamin A and RA in growth and energy balance comes from the study of photoperiodic animals such as hamsters which display increased growth and feeding under long day (summer‐like) conditions compared to short (winter‐like) daylength. Many components of the RA signalling pathway are upregulated in the hypothalamus of long day‐acclimatized rodents including the retinoic X receptor (RXR) which can heterodimerize with either the TH receptor or the RA related receptor (Helfer et al., 2012; Ross et al., 2005; Ross et al., 2004; Shearer et al., 2010; Shearer et al., 2012b). Finally, the importance of Raldh1 in energy balance is highlighted in the finding that *Raldh1* ^−/−^ mice are highly resistant to diet‐induced obesity (Ziouzenkova et al., 2007). This phenotype was ascribed to an excess of retinaldehyde in adipose tissue due to a lack of Raldh1. However, more recent studies have identified Raldh1 as the only RA‐synthesizing enzyme in the mouse hypothalamus (Helfer et al., 2012; Shearer et al., 2010) and therefore the downregulation of hypothalamic RA signalling in *Raldh1* ^−/−^ mice may also play a role in prevention of obesity.

**Figure 8:**
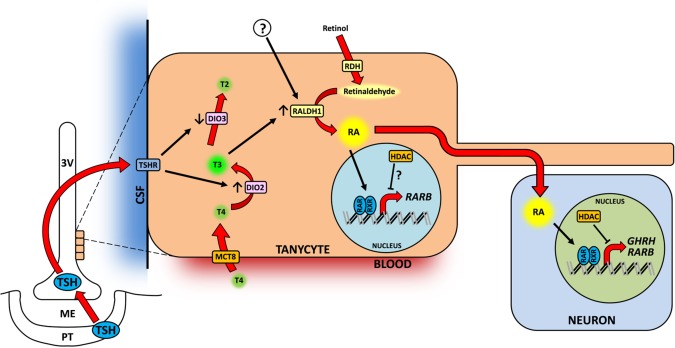
A proposed model of retinoic acid function in the hypothalamus. Thyroid hormone circulates in the blood in the form of thyroxine (T4) and is taken into hypothalamic tanycytes via transporters such as monocarboxylate transporter 8 (MCT8). T4 is converted by type II deiodinase (DIO2) to the more active triiodothyronine (T3), which is inactivated by DIO3. Stimuli such as fasting can result in a local increase in T3 in the tanycytes, upregulating *Raldh1* expression. RALDH1 synthesizes retinoic acid (RA), which enters the nucleus and upregulates expression of target genes via binding to retinoic acid receptors (RAR). Tanycytes project long processes into the parenchyma which contact hypothalamic neurons and RA may be released immediately adjacent to target cells. Histone deacetylases (HDAC) may further modulate the action of RA on its target genes. In seasonal animals, the release of thyroid‐stimulating hormone (TSH) from the pars tuberalis of the pituitary (PT) is increased in summer‐like, long‐day conditions. TSH binds to its receptor (TSHR) on the surface of tanycytes and increases DIO2 expression while supressing DIO3, and leading to an increase in T3 in the tanycytes. [Color figure can be viewed in the online issue, which is available at wileyonlinelibrary.com.]

The data presented in this study suggest that TH signalling has the potential to lie upstream of RA in the hypothalamus, as T3 upregulated *Raldh1* expression both *in vivo* and *ex vivo*. This is consistent with previous observations in VAD rats. Expression of both TRs and RARs is suppressed in the brain of VAD rats (Husson et al., 2003). Injecting VAD rats with RA only reactivated RA signalling, but T3 administration reactivates RAR and TR expression, suggesting that RA signalling can lie downstream of TH signalling in the brain. In the hypothalamus, RA is synthesized by tanycytes, the only cells in the hypothalamus that express both Dio2 and Raldh1, with Dio2 synthesizing T3, which can then act to induce *Raldh1* and increase levels of RA potentially to act on both tanycytes and neurons. This proposed pathway is illustrated in Figure 8. This system is notable for the very rapid induction of *Raldh1* transcript. Raldh1 protein is transported along the length of the tanycyte fibres (Shearer et al., 2010), which have been shown to contact Agrp/Npy neurons in the hypothalamus (Coppola et al., 2007), potentially releasing RA immediately adjacent to the target cells. This novel pathway provides a new route by which TH may promote expression of neuronal Agrp to increase appetite (Varela et al., 2012) and may also provide a mechanism by which TH could increase Ghrh to promote growth (Ross et al., 2011). The potential also exists for this to be a mechanism by which TH controls neurogenesis given the recent finding of RA's control of cell proliferation in the neurogenic regions of the hypothalamus (Shearer et al., 2012b). β2‐tanycytes have been proposed as a neural stem cell population (Lee et al., 2012) and express Fgf10 (Haan et al., 2013). This potentially influences the birth of new neurons that modulate hypothalamic control of energy balance (Kokoeva et al., 2005; Lee et al., 2012; McNay et al., 2012).

The action of Raldh1 as a downstream effector of TH was recently described in the developing mouse cerebral cortex (Gil‐Ibanez et al., 2014). Our own analysis of the mouse *Raldh1* promoter identified a putative TRE, similar to the rat. Further, non‐verified gene array analysis of hypothyroid rats, 24 hours after TH treatment, identified *Raldh1* as a putative TH‐inducible gene in the hypothalamus (Barrett et al., 2014). This same gene array analysis also identified two other unverified retinoid‐related genes potentially downstream of TH; the first *Dhrs7c* (dehydrogenase/reductase SDR family member 7C, SRP‐35) which synthesizes retinaldehyde from vitamin A, the substrate of Raldh1 (Treves et al., 2012). Also putatively identified to be strongly induced by TH was *Rpe65*, a retinoid isomerase (Kiser and Palczewski, 2010).

This study did not show an effect of T3 or RA on *Npy*/*Cart* expression. However *Npy* is presumed to be downstream of TH given that it is upregulated in hyperthyroid animals (Lopez et al., 2010). Hence, there must be alternative regulatory pathways downstream of TH signalling that control NPY, either in the hypothalamus, or extrahypothalamic. Other signalling routes may also exist for TH regulation of *Agrp*, as recently suggested to involve mTOR (Varela et al., 2012), but such pathways may set up long‐term changes in contrast to the rapid action of TH to induce *Raldh1*. That RA was found to result in a slight increase in *Pomc* expression was unexpected given that *Agrp* was increased and these factors usually display reciprocal changes. *Pomc* expression is known to be regulated by RA in the pituitary (Paez‐Pereda et al., 2001) and protein expression of adrenocorticotropic hormone (ACTH), a product of POMC, appeared increased by RA in organotypic cultures of mouse hypothalamus (Shearer et al., 2010). There are instances in which *Pomc* and *Agrp* are simultaneously increased in the hypothalamus, in conditions where both TH and RA signalling are increased (Ross et al., 2009). Further, there is some evidence that NPY suppresses *Pomc* expression via the Y2 NPY receptor (Garcia de Yebenes et al., 1995) and *in vivo* NPY may be capable of opposing the transcriptional activation of *Pomc* by RA.

Some, but not all, of the RA‐regulated genes in the hypothalamus were subject to regulation by epigenetic modifications. Epigenetic control of gene expression via histone acetylation has been previously shown to mediate, in part, some of the effects of environmental influences on the brain (reviewed by Fagiolini et al., 2009), including alteration of energy balance. For example, changes in HDAC expression and histone acetylation have been observed in the ventromedial and paraventricular hypothalamic nuclei of mice that were either fasted or fed a high‐fat diet (Funato et al., 2011). In addition, epigenetic changes in the foetal hypothalamus caused by maternal stress have been linked to long‐term alterations in energy balance, including susceptibility to diet‐induced obesity (Paternain et al., 2012; Stevens et al., 2010).

These observations suggest that epigenetic modifiers play an important role in the regulation of metabolic states, particularly in the case of persistent changes over longer timescales. There is some evidence that the RA signalling pathway is a major target of HDACIs (Epping et al., 2007). Unliganded RARα is known to suppress transcription of RA target genes by recruiting components of the corepressor complex (Hauksdottir et al., 2003), including HDACs, and HDACIs act partly via derepression of RA signalling (Epping et al., 2007). The data presented in this study, in which induction of *Rarb* and *Ghrh* by RA was potentiated in the presence of a class I/II HDACI, suggests that epigenetic regulation of hypothalamic function can, in part, act via the RA signalling pathway.

In summary, this study has demonstrated an extra step of regulatory control in the hypothalamic tanycytes which provides a mechanism by which TH has the possibility to control gene expression through sequential nuclear receptor steps, first the TH receptor followed by the RA receptor. Such a route may provide an amplification step for TH signalling. This pathway also provides a point of epigenetic regulation of hypothalamic function. In the hypothalamus, HDACs are involved in masculinization of the brain during the early postnatal period potentially through the nuclear receptor estrogen receptor α and aromatase (Matsuda et al., 2011). Control of corticotropin‐releasing hormone in the hypothalamus by the glucocorticoid receptor, another member of the nuclear receptor superfamily, is potentially mediated by HDACI (Miller et al., 2011). Epigenetic changes in the DNA methylation and histone acetylation states of the promoters of hypothalamic genes involved in energy balance, such as *Pomc* and *Npy*, have been found to result from maternal undernutrition or stress (Paternain et al., 2012; Stevens et al., 2010). This represents a mechanism by which hypothalamic plasticity may be moulded.
